# Discovering opinion leaders for medical topics using news articles

**DOI:** 10.1186/2041-1480-3-2

**Published:** 2012-03-15

**Authors:** Siddhartha Jonnalagadda, Ryan Peeler, Philip Topham

**Affiliations:** 1 Lnx Research LLC, 750 The City Drive South #470, Orange, CA 92868, USA; 2 Department of Biomedical Informatics, Arizona State University, Phoenix, AZ 85004, USA

## Abstract

**Background:**

Rapid identification of subject experts for medical topics helps in improving the implementation of discoveries by speeding the time to market drugs and aiding in clinical trial recruitment, etc. Identifying such people who influence opinion through social network analysis is gaining prominence. In this work, we explore how to combine named entity recognition from unstructured news articles with social network analysis to discover opinion leaders for a given medical topic.

**Methods:**

We employed a Conditional Random Field algorithm to extract three categories of entities from health-related new articles: Person, Organization and Location. We used the latter two to disambiguate polysemy and synonymy for the person names, used simple rules to identify the subject experts, and then applied social network analysis techniques to discover the opinion leaders among them based on their media presence. A network was created by linking each pair of subject experts who are mentioned together in an article. The social network analysis metrics (including centrality metrics such as Betweenness, Closeness, Degree and Eigenvector) are used for ranking the subject experts based on their power in information flow.

**Results:**

We extracted 734,204 person mentions from 147,528 news articles related to obesity from January 1, 2007 through July 22, 2010. Of these, 147,879 mentions have been marked as subject experts. The F-score of extracting person names is 88.5%. More than 80% of the subject experts who rank among top 20 in at least one of the metrics could be considered as opinion leaders in obesity.

**Conclusion:**

The analysis of the network of subject experts with media presence revealed that an opinion leader might have fewer mentions in the news articles, but a high network centrality measure and vice-versa. Betweenness, Closeness and Degree centrality measures were shown to supplement frequency counts in the task of finding subject experts. Further, opinion leaders missed in scientific publication network analysis could be retrieved from news articles.

## Background

We are witnessing an exponential increase in biomedical research citations in PubMed. However, Balas and Boren [1] estimated that translating biomedical discoveries into practical treatments takes around 17 years, and 86% of research knowledge is lost during this transition through peer-review process, bibliographic indexing and meta-analysis. At the other end, pharmaceutical companies spend on an average 24% of their total marketing budgets on opinion leader activities [2]. We can reduce such huge delays and costs in bringing discoveries to practice by connecting those who produce the knowledge with those who apply it. An important step in this direction is the large-scale discovery of subject experts and key opinion leaders involved in specific areas of research, based on their mentions in literature and news articles.

Public health programs manually identify opinion leaders to promote an intervention or a change in behavior and norms [3]. However, it is becoming increasingly common in the domain of medical informatics to study the interaction patterns of scientists in relation to a research area or a department using Social Network Analysis (SNA) [4,5]. Although there are systems that assign topics of expertise to the identified persons [6,7], there are no systems that identify the opinion leaders themselves. In this paper, we explore how social network analysis could be applied for studying the relative media presence of persons based on their mentions in news articles. There are several text mining systems that extract named entities such as Person, Organization and Location from English news [8-10]; Protein, Gene and other biomedical entities categories from biomedical literature [11,12], Medical problem, Treatment and Test categories from clinical notes [13,14]. Similar methods could be used to extract subject expert names from medical news articles. The scope of this work is two folds: 1) to use existing text mining methods for extracting the names of subject experts, and 2) ranking the subject experts based on their media presence using their mention frequency and network analysis metrics to find opinion leaders.

The problem of extracting the relevant concepts automatically from text is known as "Named Entity Recognition and Classification", or "Named Entity Recognition (NER)". This has been studied for almost two decades [15] and there has been significant progress in the field. Earlier attempts were predominantly dictionary or rule-based systems; however, many modern systems use supervised machine learning where a system is trained to recognize named entity mentions in text based on specific (and typically numerous) features associated with the mentions that the system learns from annotated corpora. Thus, machine learning based methods are dependent on the specific technique or implementation details and the features used for it. In the former category, generative models (e.g. Naïve Bayes Classifier and Hidden Markov Models) and instance-based classifiers (e.g. Logistic Regression and Naïve Bayes Classifier) proved to be less accurate for extracting concepts or named entities from text than sequence-based discriminative models like Conditional Random Fields [16,17]. Most of the high-performing tools use non-semantic features such as parts of speech, lemmata, regular expressions, prefixes and n-grams. The high computational cost associated with using deep syntactic and semantic features had traditionally restricted the NER systems to the orthographic, morphological and shallow syntactic features.

Normalization, on the other hand, is the step of disambiguating polysemy and synonymy. Polysemy is the phenomenon of the same name having different meanings in different contexts. For example, Dr. John Doe working in ASU on obesity might not be the same person as Dr. John Doe working at EBI even if he is also working on obesity. The first step in normalization is to assign different identifiers to polysemous entities. Synonymy is the phenomenon of two different names having the same meaning in respective contexts. For example, a scientist who worked previously in UP on clinical text mining might be the same person if she changes her last name after marriage and moves to UC to continue working on clinical text mining. Synonymous names are assigned a common identifier, after NER.

Direct ad-hoc literature searches for finding subject experts are time consuming, and rely on a researcher's library science skills and domain expertise, as well as their ability to distill massive quantities of information. Surveys are often helpful in overcoming the limitations of literature searches, as a replacement or a supplement. However, collective wisdom can be wrong [18] and non-responders might lead to bias. Alternatively, an expert familiar with the community could identify its most influential members [19]. This is known as the informant method, where individuals within a particular community name someone they believe to be influential, but not necessarily someone who influences the informant. Arguably, the expert's bias leans towards the more visible and higher profile organizations. The self-identification method assists in assessing entity's impressions of themselves as key players. Most people view their own work as important, and as a result, may estimate themselves to be more important and influential than they actually are. The informant approach is reasonable for small, relatively homogeneous communities where informants are likely to have knowledge of the entire community, but not so when the community has thousands of members [20]. With SNA, it is possible to analyze a much larger social network containing thousands of nodes. For example, a cross-sectional study of the spread of obesity used 12,067 nodes and 38,611 links [21]. With such a large, objectively gathered sample, we might reduce the bias significantly.

For this study, we chose obesity as the topic and obtained news articles related to this subject from the Internet. The links were provided to us by Intuli (http://intuli.com) using their proprietary technology that uses archived news articles and keywords related to "obesity" (see Additional file 1 for the links). After extracting Person, Organization and Location concepts from the media articles, we applied an approach to identify subject experts among persons by filtering out persons without relevant education or affiliation and without scientific publications. We then performed social network analysis using the identified subject expert mentions in the sample news articles related to obesity.

## Methods

**Figure 1:**
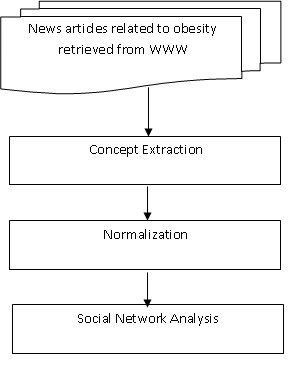
**Overall architecture**. We first retrieved the articles related to obesity from the Internet using web-crawlers. The Person, Organization and Location named entities were extracted from the collected articles. Among the person names, only medical experts were retained. The semi-automatic normalization step addressed polysemy as well as synonymy. In the social network analysis step, we analyzed the network presence of the subject experts.

Figure 1 describes our process to create a social network for subject experts. It constituted of extracting concepts, filtering the names of subject experts, manually normalizing subject expert mentions and using SNA to identify opinion leaders. We conducted our research in relation to the disease area of obesity. However, this process is applicable to any medical topic.

### Concept extraction

**Figure 2:**
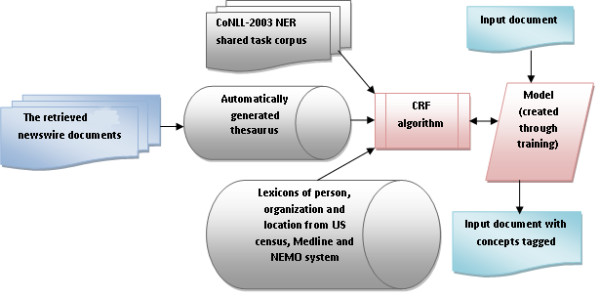
**Concept extraction process**. The CRF system is trained using the CoNLL-2003 NER shared task corpus and run on the 147,528 obesity-related news articles. The model created during the training phase is used to tag the input sentences with the concepts "person", "organization" and "location".

Figure 2 describes our machine learning system to extract the names of person, organization and location. We used CoNLL-2003 NER shared task corpus for English documents labeled with Person, Organization and Location along with other named entity classes [22] for training, and the retrieved news articles (see Additional file 1) for testing i.e. the execution of the trained model. "Boilerplate program" [23] converted the html format of the news articles to text format.

We used the first order CRF algorithm as implemented by MALLET [24]. Concepts are identified by tagging tokens of each sentence with labels to represent whether the token belongs to each concept class (inside) or not (outside). More sophisticated labeling also identifies whether a token begins or ends a concept class. Previous work (e.g. [25]) has shown that the accuracy is similar for all label types such as - IO, IOB and IOBEW, where I stands for labeling a token to be Inside, O for Outside, B for Beginning, E for End and W for Within. A CRF-based system calculates the probabilities of different labeling sequence assignments for sentences based on the individual words (tokens) using their natural language (i.e. text) features in relations to the words in the training sentences. It chooses the sequence of labels for all the concepts with the highest probability. The time complexity of the CRF algorithm is O(L^2^*N*M*F*I), where L is the number of labels, N is the number sentences, M is the average length of the sentences, F is the average number of the features and I is the number of iterations. Hence, we chose the IO notation that allows minimum labels for labeling to minimize time complexity. Thus, Iperson, Iorg, Iloc and O are the labels used because Person, Organization and Location are the annotated concepts.

Table 1 describes the features used for the CRF algorithm. The feature extraction component extracts features of natural language at the level of words (lexical), syntax, context (pragmatic) and meaning (semantic). The features (other than distributional semantic features, see below) are adapted from BANNER [25], an open source NER system. We compiled the dictionaries for person names from the US census and names of authors in Medline. The dictionaries for organizations and locations are reused from the "NEMO: Normalization Engine for Matching Organizations" [26] project. It was shown previously that the meaning of words could be represented in high-dimensional vector space. Semantic vector representation [27] of terms are created to automatically obtain a thesaurus of terms that are paradigmatically similar (occur in similar contexts; see [13,28] for more information). The Dragon toolkit [29] tagged the part of speech for each word in a sentence. The other features are generated using regular expressions and simple rules.

**Table 1:** List of features used in the CRF method.

Feature name	Type	Description
Dictionary	Semantic	Person names; Organization names; Location names

Distributional	Semantic	Distributional thesaurus

Section	Pragmatic	Name of the section in which the sentence appears

Part of speech	Syntactic	Part of speech of the token in the sentence

Others	Lexical	Lower case token, Lemma, Prefixes, Suffixes, n-grams, Matching patterns such as beginning with a capital, etc.

Dictionary features: all the three dictionaries contain words that have a single token and are obtained by removing stop words. Each dictionary corresponds to one feature depending on whether a token is present in the dictionary. Distributional features: using the Semantic Vectors package [27] trained on the text retrieved from the links obtained for the case study, each word is represented in a 2000-dimensional vector space. The vector representation is used to find the 20 most similar words from the text to each word. For each token, we thus have 20 distributional semantic features that represent the entries in the thesaurus. Section features: section names are detected automatically using simple rules (e.g. a sentence ending with a semi-colon). Other features: there are about a hundred more features considering different part of speech tags according to Penn Treebank format, the different matching patterns used, prefixes, n-grams etc

### Filtering out likely irrelevant person names

The CRF classification algorithm, because of the inability to perform nested labeling, does not label names of people within an organization name. However, when the features are not strongly indicative that a phrase belongs to an organization, it might label a person within the phrase. For example, the second *Mayo *in "*Mayo Clinic is a leading hospital for Obesity. So, I visited Mayo to know more about Obesity*", could be tagged as a Person. Hence, the system removes person names that are part of a major organization name after the annotation by the CRF classifier.

Since research work is an important identifier of expertise, the persons who have no published work are not considered as subject experts since they are not likely to be authorities on the subject. Such names are eliminated by further constraining that the person names should be within 100 characters (in any direction) of certain keywords indicating that they are likely to be scientists or closely associated with biomedical research. The complete list of the keywords that include acronyms such as Dr, MD and PhD is presented in Table 2. To further aid in this, we counted the number of their publications indexed in PubMed (using their first and last name) and persons having fewer than 10 publications are removed.

**Table 2:** Keywords used to filter subject expert mentions out of person mentions in news articles

Dr	MD	PhD	M.D
PhD	Prof	Dr.	M.D.

Ph.D.	Prof.	Program Director	Professor

Journal	Colleague	Colleagues	Researcher

Faculty	Doctor	Doctors	Publish

Published	University	Hospital	Hospitals

Research	Lab	Laboratory	School

Engineering	Sciences	Institute	Institutes

Clinic	College		

### Normalization

The names of extracted subject experts were checked manually for polysemy and synonymy using the assistance of a heuristic rule-based system that takes into account the lexical distance between two person names, their associated organization and their location. The matching engine was developed at Lnx Research to support resolving more quickly the issues caused by polysemy and synonymy. It exploits known likelihoods of common co-authors and common organizations or locations as well as lexical distance between named entities. While this can be done automatically with high precision for most person names, the rest needs to be matched manually. We estimate that the accuracy of this proprietary system is greater than 95%. As a result of this step, a list of unique names of (potential) subject experts is generated.

### Social network analysis

We generated links between subject experts (persons whose names are extracted by the previous steps) if they are mentioned in the same news article. The resultant co-mention network is analyzed using traditional social network analysis techniques: Degree centrality, Betweenness centrality, Closeness centrality and Eigenvector centrality. In social network terms, these centrality measures are associated to prestige, power, prominence, and importance, respectively - sometimes called the four P's [30]. Degree centrality, the number of nodes immediately connected to a node, suggests the expert node has more prestige than comparable nodes. This is particularly evident in friendship networks where linkages represent friendship between people. For example, a famous person in Facebook may have hundreds of thousands of friends - an amount considerably greater than the typical Facebook account holder. Betweenness centrality relates to the node's importance in connecting and transmitting information across the entire network. Closeness reflects a node's position relative to the geodesic center of a network. Nodes close to the center are prominent. As an example, consider the typical club or professional organization. The key members (President, Vice President, Membership Chairperson, Activities Chairperson, etc.) are all central and prominent to the group's functioning. The fourth measure, Eigenvector centrality, is most analogous to importance. In this measure, consideration is given to the connections of a node's connections, or in the Facebook example, your friends' friends. A person with connections to people with few friends is different from a person with the same number of connections to friends with many friends themselves. The first three aforementioned centrality measures are more completely discussed by Freemen [31] and the fourth measure, Eigenvector by Bonacich [32].

Formally, for a graph (*V*, *E*) with *n *vertices [33,34],

- the Degree centrality *C_D_*(*v*) for vertex *v *is:

$$C_{D}\left(v\right)=\frac{\deg\left(v\right)}{n-1}$$

where deg(*v*) is the number of edges connected to *v*.

- the Betweenness centrality *C_B_*(*v*) for vertex *v *is:

$$C_{B}\left(v\right)=\underset{s,v,t\in V\;and\;s\neq v\neq t}{\sum }\frac{\sigma_{st}\left(v\right)}{\sigma_{st}}$$

where σ*_st _*is the number of shortest paths from *s *to *t*, and σ*_st_*(*v*) is the number of shortest paths from *s *to *t *that pass through a vertex *v*;

- the Closeness centrality *C_C_*(*v*) for a vertex *v *is:

$$C_{C}\left(v\right)=\frac{1}{\sum_{t\in V-v}d_{G}\left(v,t\right)}$$

where d_G_(*v*, t) is the shortest distance between *v *and t;

- the Eigenvector centrality *C_E_*(*v*) for a vertex *v *is calculated recursively using the Eigenvector centrality values of the adjacent vertices:

$$C_{E}\left(v\right)=\frac{1}{\lambda }\underset{\mu \in M\left(v\right)}{\sum }C_{E}\left(\mu \right)$$

where *M*(*v*) is the set of nodes that are connected to node *v *and *λ *is the largest eigenvalue of the adjacency matrix representing the corresponding graph.

## Results

### Concept extraction and normalization

We extracted 734,204 person mentions from 147,528 news articles related to obesity from January 1, 2007 through July 22, 2010. Of these, 147,879 person mentions have been marked as subject experts after the filtering step (using advanced degree (MD or PhD or equivalent) and by presence in PubMed). During the normalization process, we identified the mentions of the subject experts that refer to the same individual. The 147,879 subject expert mentions were mapped to 16,416 unique individuals. In addition, we extracted 834,423 organization mentions and 564,262 location mentions, which were not normalized.

For the purpose of evaluating the accuracy of our concept extraction system, we randomly chose 100 news articles and annotated the persons mentioned in the articles. As a baseline, we considered a dictionary-based system that identifies person names using a list of first and last names gathered from the US census data. Table 3 shows the performance of our machine-learning system compared to the baseline before filtering. The accuracy of the system is measured using the percentage of person names in the gold standard that were extracted (recall) and also the percentage of extracted entities that were actually person names (precision). The harmonic mean of precision and recall (F-measure) was also used to tradeoff between precision and recall. Although the recall of the baseline system is comparable for both exact and partial match of the names with the gold standard names, the precision of the machine learning system is significantly better. This is because the machine learning system is trained to learn the context from examples. The overall accuracy of the system (including filtering) for exact match is 88.5%. The precision of the system was further improved after removing the person names that are part of major organization names (data not shown).

**Table 3:** Performance of the CRF-based concept extraction system, compared to the dictionary-based baseline on 100 news articles

	True Positives	False Negatives	False Positives	Recall	Precision	F-measure
Machine learning system						

Exact Match	54	9	5	85.7	91.5	88.5

Partial Match	55	8	4	87.3	93.2	90.1

Dictionary Baseline						

Exact Match	45	18	254	71.4	15.0	24.9

Partial Match	58	5	241	92.1	19.4	32.0

Among the top 100 person names in terms of the number of mentions extracted from all the news articles, only one name was a false positive subject expert because of name ambiguity. However, the number of articles for the subject expert with that name is adjusted during the normalization step. On the other hand, the number of mentions of the top 100 persons that were extracted with the subject expert filter was 3,813, while the number of mentions without the filter was 4,572. Thus, 16.6% of mentions were filtered out because not all subject expert mentions were surrounded by the keywords.

### Social network analysis

**Figure 3:**
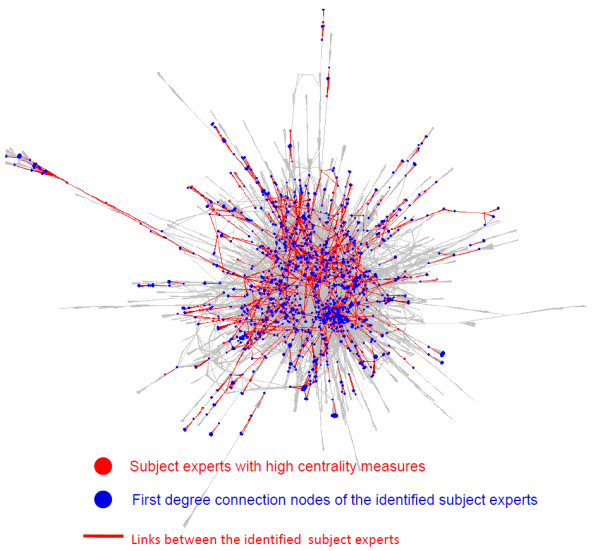
**Network map of the largest connected component with subject experts marked**. Each person appears in at least one news article. The persons appearing at the center have a higher centrality. The links are unweighted and show the co-occurrence (mentioned together in an article) of subject experts.

We constructed a network that contained 16,416 unique subject expert nodes and 97,516 links between them. The frequency of the person names as well as the centrality metrics were used to produce a list of subject experts ranked by their relative importance. Figure 3 shows the largest connected component (with 11,742 nodes and 56,431 links between them) among the subject experts extracted and connected for this study. The fact that more than half of the subject experts are in the largest connected component signifies that the persons in this network are well connected. Many of the subject experts are at the center of the network, which shows their high connectedness with other persons in the network.

Using the social network analysis package in R [35], the key network metrics were calculated for all individuals in the largest connected component. We found that a majority of these experts are prevalent closer to the center of the network. This is where the Betweenness centrality, Eigenvector centrality, and Closeness centrality are high. We found that subject experts toward the periphery connect entire branches or arms of the structure to the center. These arms may be based on specialty in research, geography, institution, or some other cause. Experts that connect branches have a high Betweenness score, while Closeness scores can remain relatively low and the prestige and importance of a person in the network still remains high. These features contribute to a kind of fingerprint of a person's functional role in a network. A person with a high Betweenness score, medium Eigenvector score, and a low Closeness score may be the best choice for communicating information to an arm of a network. This logic extends to other parts and features of the network. If the purpose of a news piece is to raise awareness outside the community, subject experts with high Eigenvector scores but low Betweenness scores and medium Closeness scores may be the most effective to disseminate the message.

We have created a list of 51 subject experts that rank among the top 20 in at least one of the metrics used: the number of mentions in the news articles, Degree centrality, Betweenness centrality, Eigenvector centrality and Closeness centrality. Manual evaluation based on the information available on the Internet revealed that many won awards for teaching and research, published book chapters and authored popular bestselling books on topics related to obesity. In some cases, they have been national newsmakers because of innovative revolutionary research in obesity. We found that 41 among the 51 (80.4%) could be considered as opinion leaders in obesity. Only four of the persons that rank among the top 20 by number of mentions in the news articles were not considered opinion leaders in obesity. Two of them are (neuro and cardiothoracic) surgeons who corresponds to media on a variety of health problems; two are social networks researchers who use obesity as an example in their research. We noticed that three of these four researchers were not among the top 20 in any of the SNA metrics. The full evaluation details are available in Additional file 2.

We then used the presence of a person's biography in Wikipedia as an objective measure of a person's expertise and media presence. Although Wikipedia is an open encyclopedia, only biographies that are "significant, interesting, or unusual enough to deserve attention or to be recorded" are present [36]. Among the 51 persons that were among top 20, in at least one of the metrics, 27 have biographies in Wikipedia. However, Wikipedia does not always index a prominent subject expert. For example, as of July 31 2011, David Haslam (the chair of National Obesity Forum of UK) and Barry Popkin (a US-based anti-obesity crusader since the last three decades) who rank high in our list did not have a biography in Wikipedia. Twelve of the top 20 persons in Betweenness, Closeness and Degree centralities each have Wikipedia biographies. Eleven of the top 20 persons in number of mentions in news articles are mentioned in Wikipedia. However, only seven of the top 20 persons in Eigenvector centrality have Wikipedia biographies. While Wikipedia itself is not exhaustive and hence not useful for ranking or even simply listing subject experts, we used it as an external validation for our method.

Among the top 20 subject experts ranked by the number of media mentions, all have contributed significantly (20 or more peer review journal articles) to both the advancement of science in the literature (mean = 117 publications) and in newswire (mean = 56 media mentions). Twelve of these 20 have top 20 Betweenness scores and nine of the top 20 have top 20 Closeness scores. Among the different SNA metrics, the Eigenvector centrality is the least useful based on the external evaluation.

We have also created a list of the most frequently extracted subject experts from media that are highly relevant to the subject, but were missed using co-authorship information used in our previous work [37]. 34 out of the top 100 (by news mentions) subject experts are not part of the largest connected component of the collaboration network based on obesity publications. A likely reason is that some opinion leaders do not publish in scientific journals, but are active in educating the public and appearing in media. This suggests that news articles can complement authorship information in scientific databases in the identification of subject experts.

## Discussion

The named entity recognition or concept extraction component uses the Conditional Random Field algorithm which is currently used in some of the best performing systems in NER [9,12,13,25,38]. Based on the performance in the randomly created gold standard, we estimate that the accuracy of the system for extracting person names is between 85-90% measured using F-score, where F-score is the harmonic mean of precision (about 90%) and recall (about 85%). Creating the CRF model or classifier, a one-time process, took around 10 hours. The various concepts were extracted within an hour using Hadoop [39] data-processing framework: the process was concurrently executed on the 147,528 links using a leased cloud of 20 octa-core servers each having 15 GB of RAM.

We currently use heuristics such as presence of keywords and publication counts to retain subject expert names among the extracted person names. In the future, we could use an instance classification algorithm such as Support Vector Machines with the orthographic features as well as publication counts to create a classifier that automatically separates subject experts from person names. The data we are currently gathering for the obesity project as well as future projects will be used as training data.

The analysis of the network of subject experts revealed that a subject expert might have fewer mentions in news articles. The Eigenvector centrality is found to be the least useful metric, but different metrics tend to find different persons.

The extrapolation of these findings can help us differentiate the way subject experts can be interpreted. This depends on the end user classification of a subject expert. A celebrity can lend their name to a social cause for public awareness in a particular disease area but that does not qualify them to be an expert in that area. The media count in those cases might be higher but that will be due to higher mention of the name in electronic media. It does not necessarily correlate with the deemed expertise on that particular topic. For a media-focused or a consumer goods company, a person with a high media count might simply make the mark due to the ease of name identification with the general public.

## Conclusion

The major contribution of this study is to use named entity recognition (concept extraction) for discovering potential opinion leaders based on mentions in news articles. This provides a platform to "create" a list of prominent subject experts empirically using publicly available text. Additionally, we learned that network centrality measures supplement frequency counts in finding opinion leaders with media presence. Among the 51 subject experts that are among top 20 in at least one of the metrics we have used, 41 were considered as opinion leaders for obesity. Betweenness, Degree and Closeness centrality metrics are at least as accurate as the frequency count. The combination of subject experts that rank high in network centrality measures in additions to the number of mentions gave a list of the relevant opinion leaders to obesity. Further, a significant number of opinion leaders were discovered from news articles that were not discovered in our previous work using PubMed data. Network analysis of person names in news articles is useful as a supplement to the number of news articles citing a person in understanding the relative media presence of persons for a medical topic. Identical work needs to be conducted in other disease areas to validate further the model and the findings presented here.

## Competing interests

The authors declare that they have no competing interests.

## Authors' contributions

SJ and PT designed the system. SJ implemented the system in Java and wrote the manuscript. RP provided assistance in the qualitative analysis of the results. All authors read and approved the final manuscript.

## Availability of supporting data

The links to the internet news articles used in the study is available as part of this paper as Additional File 1. A Table that lists the relative centrality measures of the identified opinion leaders, their presence in the Wikipedia, their mention frequency in the news articles and their publication counts in the disease area is available as Additional File 2. The names of the persons are anonymized. The software used to generate this data uses third-party proprietary components and is not shared, but might be available at no cost as a web-service for non-commercial research projects. Please contact the corresponding author or email info@lnxresearch.com.

## Supplementary Material

Additional file 1 **Links to the articles used in the study**. The file lists all the article links related to obesity that were provided by Intuli (http://www.intuli.com). Click here for file

Additional file 2 **Centrality measures and other comparison of the identified opinion leaders**. This table lists the relative centrality measures of the identified opinion leaders, their presence in the Wikipedia, their mention frequency in the news articles and their publication counts in Obesity. Click here for file
